# Percutaneous Treatment of Mitral Paraprosthetic Regurgitation: an Alternative to Surgery

**DOI:** 10.5935/abc.20150115

**Published:** 2015-10

**Authors:** Roney Orismar Sampaio, Alessandra Gomes de Oliveira, George Barreto Miranda, Pedro Alves Lemos Neto, Marcelo Luiz Campos Vieira, Flávio Tarasoutchi

**Affiliations:** Instituto do Coração do Hospital das Clínicas da Faculdade de Medicina da Universidade de São Paulo (USP), São Paulo, SP – Brazil

**Keywords:** Mitral Valve / surgery, Heart Valve Prosthesis Implantation, Prosthesis-Related Infections / complications, Heart Failure, Hemolysis, Atrioventricular Block

## Introduction

Paraprosthetic regurgitation occurs in approximately 7%–17% of patients undergoing mitral valve replacement and 2%–10% of patients undergoing aortic valve replacement^1,2^. Typically, this regurgitation is discrete and does imply major clinical complications; however, when it is moderate or severe, the consequences can be serious, leading to heart failure and/or hemolysis^3,4^. It is estimated that approximately 1%–5% of cases develop to more clinically severe conditions^1,4^. The main causes of paraprosthetic regurgitation are calcification of the valvular annulus, infection, suture technique, and size and shape of the prosthesis^1,5^.

Usually, surgical treatment is considered as the first treatment option for symptomatic patients^4^. In 1992, however, an alternative treatment via percutaneous occlusion of the paravalvular orifice was proposed for cases in which the patient faces high risk from surgical treatment^6-8^.

## Case Report

A 70-year-old male patient received a mechanical mitral prosthesis implant in 2002 during his fourth heart surgery. At that time, the patient presented multiple postoperative complications: septic shock requiring high doses of vasoactive medications, acute renal failure and atrial fibrillation, and prolonged hospitalization. After this period, the patient made good progress and had no limitations on daily activities, until 3 years previously, when he began to experience recurrent hematuria due to intravascular hemolysis. The patient was clinically followed until June 2012, when the hemolysis markedly worsened, in association with heart failure up to functional class III [New York Heart Association (NYHA)]. On physical examination, there was a regurgitant systolic murmur +++/4 in the mitral area and crackle at the base of the lungs.

**Figure 1:**
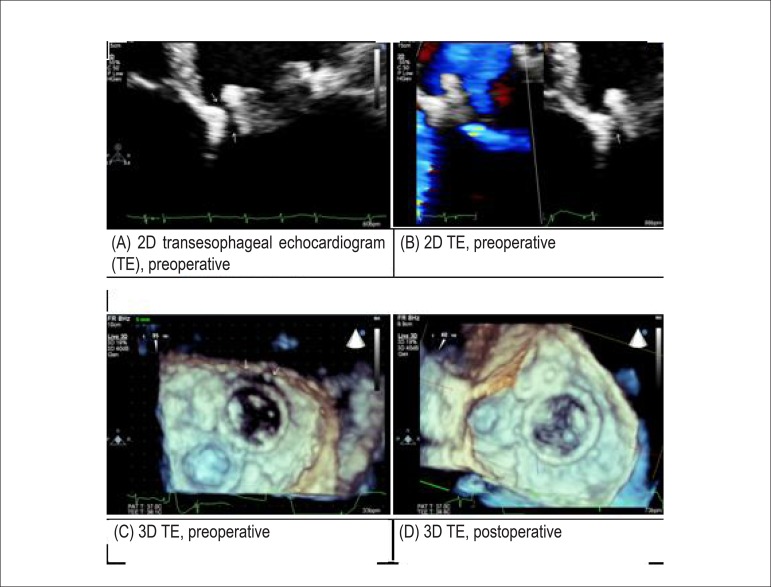
(A) Two-dimensional transesophageal echocardiogram (2D-TEE). (A) Preoperative 2D-TEE (B and C); (C) preoperative 3D-TEE; (D) post-operative 3D-TEE

Laboratory examinations showed the following: lactate dehydrogenase (LDH) 3256 U/L [reference value (RV): 85‑227 U/L], haptoglobin 0.2 g/L (RV: 0.3–2.0 g/L), hemoglobin (Hb) 7.3 g/dL (RV: 13.0–18.0 g/dL), hematocrit (Hct) 22% (RV: 40.0-52.0%), and hemoglobinuria. Two‑dimensional transesophageal echocardiography demonstrated the mechanical prosthesis with normal mobility for its elements as well as moderate/significant periprosthetic regurgitation (Figures 1A and B) associated with maximum LA–LV diastolic gradient estimated at 13 mmHg (average, 4 mmHg). The valve area was estimated at 3.2 cm^2^. Because of the difficulty in accurately determining the size of the regurgitant orifice and subsequently choosing the best occlusion device, we performed three-dimensional transesophageal echocardiography. This technique identified both anterior and posterior periprosthetic insufficiency: anterior gap of 22 mm along the long axis and posterior gap of approximately 12 mm (Figure 1C). Because of the risk of many postoperative complications (as in 2002) and the high surgical risk associated with a fifth heart surgery, in a joint decision along with the patient and family, we opted for percutaneous treatment.

### Treatment

**Figure 2:**
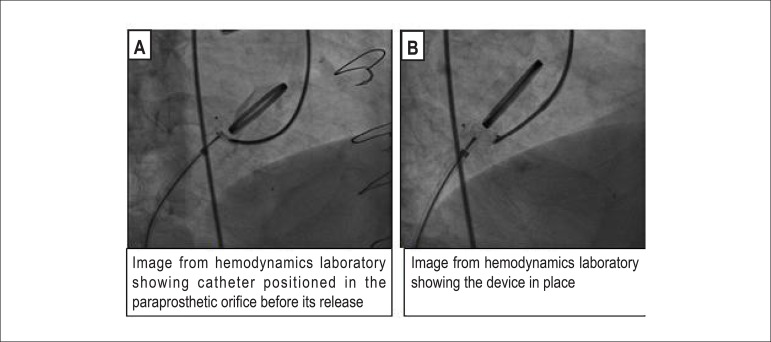
(A) Hemodynamics laboratory image of the catheter in the paraprosthetic orifice before deployment. (B) Hemodynamics laboratory image of the deployed device.

Two percutaneous devices, “Duct Occluder 8 mm × 6" and “5 mm VSD," were implanted, with a significant reduction in postimplant regurgitation (there was a decrease of approximately 70% in the anterior orifice) (Figure 2). A second 3D echocardiography (Figure 1D) was performed 45 days after the procedure and demonstrated good positioning of the occlusion devices and minimum residual paraprosthetic reflux. Six months after the procedure, the patient presented with a second-degree atrioventricular block, Mobitz II, and two episodes of presyncope; a DDD pacemaker was implanted. The patient improved to functional class I (NYHA). Physical examination at this time showed a slight systolic murmur of mitral regurgitation (+/4) and improved laboratory results (LDH 728 U/L, Hb 10.5 g/dL, Hct 32.3%).

## Discussion

Paraprosthetic reflux is a significant complication of valve replacement surgery. Percutaneous closure of a paraprosthetic orifice is now considered a safe procedure, providing an alternative to surgery in patients with a high surgical risk. However, there are still few reports of percutaneous intervention in patients with paraprosthetic regurgitation.

In 2006, Pate et al.^9^ published a study of 10 patients who were not candidates for surgery and underwent percutaneous closure of mitral paravalvular leak; this author noted a 70% success rate for this procedure, associated with clinical improvement. However, four patients required a second intervention.

In 2011, Sorajja et al.^10^ published a study of 115 patients who underwent the percutaneous procedure, with technical success in 77% of cases and clinical improvement in 67%. The total number of complications 30 days after the procedure was 8.7%: death in two cases (1.7%), stroke in three cases (2.6%), vascular complications in one case (0.9%), hemothorax in four cases (3.5%), and emergency surgery in one case (0.9%).

In addition, in 2011, Ruiz et al.^8^ showed that in retrospective analysis of 43 patients, procedural success was observed in 86% of cases and clinical improvement was observed in 77%.

In this present case, 6 months after percutaneous treatment, the patient developed atrioventricular block and required a pacemaker. After an extensive review of the literature, we found no reports of second-degree atrioventricular block as a late complication of percutaneous closure of the paraprosthetic mitral valve orifice^1^. Even in the aortic position, where it may be more common considering the anatomy of the conduction system, there are no reports of this complication. The risk of this event (late implant pacemaker for AVB) seems to have been random, particularly in closing the paraprosthetic mitral orifice; however, it cannot be ruled out. Nevertheless, the fact the pacemaker was implanted 6 months after the procedure is also relevant, which in our view leaves it unclear whether a possible complication exists and is yet to be described. Therefore, this event should be noted and followed in the literature.

Therefore, we conclude that a percutaneous procedure to correct paraprosthetic regurgitation is feasible in patients with a high surgical risk and has a significant clinical impact.
